# Machine Fault Detection Using a Hybrid CNN-LSTM Attention-Based Model

**DOI:** 10.3390/s23094512

**Published:** 2023-05-05

**Authors:** Andressa Borré, Laio Oriel Seman, Eduardo Camponogara, Stefano Frizzo Stefenon, Viviana Cocco Mariani, Leandro dos Santos Coelho

**Affiliations:** 1 Automation and Systems Engineering, Federal University of Santa Catarina, Florianópolis 88040-900, Brazil; 2 Graduate Program in Applied Computer Science, University of Vale do Itajai, Itajai 88302-901, Brazil; 3 Industrial and Systems Engineering Graduate Program, Pontifical Catholic University of Parana, Curitiba 80215-901, Brazil; 4 Digital Industry Center, Fondazione Bruno Kessler, 38123 Trento, Italy; 5 Department of Mathematics, Computer Science and Physics, University of Udine, 33100 Udine, Italy; 6 Department of Electrical Engineering, Federal University of Parana, Curitiba 81530-000, Brazil; 7 Mechanical Engineering Graduate Program, Pontifical Catholic University of Parana, Curitiba 80215-901, Brazil

**Keywords:** electrical machines, empirical wavelet transform, fault detection, Savitzky–Golay filter, temporal fusion transformer

## Abstract

The predictive maintenance of electrical machines is a critical issue for companies, as it can greatly reduce maintenance costs, increase efficiency, and minimize downtime. In this paper, the issue of predicting electrical machine failures by predicting possible anomalies in the data is addressed through time series analysis. The time series data are from a sensor attached to an electrical machine (motor) measuring vibration variations in three axes: X (axial), Y (radial), and Z (radial X). The dataset is used to train a hybrid convolutional neural network with long short-term memory (CNN-LSTM) architecture. By employing quantile regression at the network output, the proposed approach aims to manage the uncertainties present in the data. The application of the hybrid CNN-LSTM attention-based model, combined with the use of quantile regression to capture uncertainties, yielded superior results compared to traditional reference models. These results can benefit companies by optimizing their maintenance schedules and improving the overall performance of their electric machines.

## 1. Introduction

Given the potential for significant reductions in maintenance costs, increased productivity, and reduced downtime, predictive maintenance of electrical machinery has become a top priority for companies [1]. Over the last few years, there has been much attention to applying predictive maintenance methods to predict electrical machine breakdowns by locating anomalies [2]. Identifying anomalous behavior in equipment is increasingly recognized as a crucial factor in anticipating maintenance actions [3] and achieving gains by avoiding unplanned downtime [4].

This paper thoroughly examines this critical topic by focusing on predicting electrical machine failures by examining time series data collected from sensors attached to the electrical machines. Optimizing maintenance schedules, increasing equipment lifespan, and enhancing the overall performance of electrical machines are some of the objectives of this study. Maintenance optimization is increasingly being explored using deep learning models [5,6,7,8], which is the focus of the method presented in this paper.

A major component of predictive maintenance is anomaly detection, which enables businesses to spot possible breakdowns quickly [9]. Time series data, such as the data gathered for this study, is particularly well suited for this kind of analysis since it enables us to look at how a given statistic changes over time [10]. Quantile regression, a statistical technique, was used to handle uncertainties in the time series data [11].

Real-world time series data generally exhibit non-linearities [12], making it challenging to apply conventional prediction techniques [13]. Therefore, advanced techniques were employed to address this issue, including convolutional neural network (CNN) [14], long short-term memory (LSTM) attention [15], and quantile regression [11], to accurately predict machine failures and manage uncertainties present in the data.

In this paper, we propose a novel approach to predicting electrical machine failures by forecasting possible anomalies in the data. Specifically, we utilize time series data from a vibration sensor attached to a real electrical machine, measuring variations in three axes (axial, radial, and radial X). By extracting features from the data using a CNN to predict time series data using a hybrid model based on LSTM with an attention mechanism, this paper presents a solution that can be applied to time series anomaly prediction that can be extended to other engineering fields.

Based on a hybrid CNN-LSTM attention model, an anomaly detection algorithm called empirical-cumulative-distribution-based outlier detection (ECOD) is applied to leverage the predictions in the 10% and 90% quantiles, providing the machine operator with the probability levels of faults. The resulting neural-based predictive maintenance tool can help companies make informed decisions about their maintenance processes.

This paper has the following contributions to improving fault detection based on time-based analyses:

- The hybrid LSTM-CNN architecture with attention and gated residual networks (GRN) enhances the accuracy of the predictions.
- The quantile regression at the network output helps to manage uncertainties present in the data.
- The use of empirical wavelet transform and the Savitzky–Golay filter assist in reducing noise in the signal and extracting relevant features for the analysis.

The remainder of this paper is organized as follows: Section 2 presents a review of the related work in predictive maintenance for electrical machines. Section 3 overviews the proposed methodology, including data collection and pre-processing, the custom hybrid CNN-LSTM attention model, quantile regression, and the ECOD anomaly detection algorithm. Section 4 presents the experimental results and analysis of the proposed approach, and Section 5 concludes the paper and discusses future research directions.

## 2. Related Works

There is a growing effort to improve ways of diagnosing electrical machines [16]; in this context, several approaches have been used to predict engine failures based on time series data. One is to use vibration analysis techniques to detect changes in the vibration signature of an engine [17], which can indicate misalignment, excessive wear, or other mechanical problems. Machine learning algorithms [18], such as decision trees [19], can be used to identify patterns in sensor readings and make predictions based on this information, while deep learning techniques have been widely used [20].

Time series spectrum analysis can be used to identify changes in the machine’s electrical signals, which may indicate failures in internal components such as bearings or windings [21]. Signal processing algorithms, such as the Fourier transform, can extract relevant information from these electrical signals and predict potential failures [22]. Furthermore, using time series forecasting, the increase in the number of failures can be monitored to assess the condition of the system being monitored [23].

State-of-the-art techniques have been applied to improve the prediction capability, such as the attention mechanism combined with AdaBoost proposed by Long et al. [24] for machine fault diagnosis. Yang et al. [25] proposed an ensemble empirical mode decomposition (EEMD) for the fault diagnosis of asynchronous machines, showing that their approach had a recognition rate of 99%, considering broken rotor bars, air gap eccentricity, and normal state.

A wide range of models have been successfully applied in time series forecasting. However, choosing the appropriate model is a challenging task [26], considering that the characteristics of the data influence model performance and since some methods have specific properties that can be helpful for non-linear forecasting. For improved signal analysis with non-linearity, techniques such as seasonality decomposition [27], wavelet transform [28], and empirical wavelet transform [29] show promise for denoising.

Hybrid models which combine noise suppression methods such as seasonal decomposition or wavelet transforms with forecasting models have been increasingly employed [30]. The advantage of using these approaches is that high-frequency variations are disregarded. The model has more effective results because it focuses on the variation trend and not on the signal noise [31]. An important observation to consider is that the filters cannot be too coarse to reduce all the variation in the signal, so a proper case evaluation must be performed [32].

Regarding noise reduction, Faysal et al. [33] proposed a noise-eliminated ensemble empirical mode decomposition (NEEEMD) method for fault diagnosis in rotating machinery. They proved that the NEEEMD could be more generalized and robust for the problem. In addition, using an ensemble-based method with wavelet packet transform (WPT), Chui et al. [34] showed that the signal-to-noise ratio could be improved using an optimized ensemble empirical model combined with WPT.

A technique that has been highlighted for noise reduction in time series forecasting is the empirical wavelet transform (EWT) [35]. Zhao et al. [36] and Xu et al. [37] applied the EWT considering an adaptive spectrum segmentation for the improvement in signal processing and fault diagnosis. Fault detection using EWT has proven to be promising, as presented by Xin et al. [38] for rotating machinery and by Xu et al. [39] for rolling bearings. Deng et al. [40], and Huang et al. [41] applied the EWT for machine bearing fault detection. The application of EWT for failure diagnosis extends to other types of machines, such as wind turbines [42], and other forecasting applications [43].

Among the time series forecasting models, there are several approaches such as neuro-fuzzy systems [44], autoregressive integrated moving average (ARIMA) [45], LSTM [46], ensemble learning methods [47], and TFT [48]. According to Li et al. [49], the TFT can improve the reliability and compactness of the forecasting and can even be applied to medium-term hourly time series data.

The wavelet neuro-fuzzy method was used by Stefenon et al. [50], who focused on time series forecasting to propose a model and assess solar prediction capability. Wavelets were incorporated into the model for feature extraction, where they analyzed whether it is possible to anticipate the production of electrical power with a hybrid model while taking solar trackers into account with a sufficient degree of precision. A forecast can be made and it can be decided whether using solar tracking is worthwhile by assuming a hybrid computational model.

A novel hybrid model that considers the benefits of linearity and non-linearity, as well as the effect of manual operations, was proposed by Fan et al. [51], combining the LSTM and ARIMA models. The LSTM model clearly outperforms the ARIMA model regarding fluctuating non-linear data. Results from coupling models outperform separate ones, with the ARIMA-LSTM model performing even better when production is adversely affected by frequent manual procedures.

Feng et al. [52] used an enhanced TFT prediction model to supply air temperatures in high-speed train carriages. The model effectively outperformed seven prominent methods in time series computing tasks, as shown by empirical simulations using a dataset comprising high-speed rail air-conditioning operations at a specific site in China. The focus of the prediction problem in the time dimension was also examined.

By combining machine learning classifiers with the feature extraction method wavelet scattering transform (WST), Toma et al. [53] proposed a system for classifying bearing faults. The experimental results showed that WST might improve bearing fault classification accuracy when compared to EWT, information fusion, and wavelet packet decomposition, achieving good classification accuracy for the fault diagnosis of rotating machinery.

To diagnose bearing faults, Van et al. [54] proposed the particle swarm optimization least-squares wavelet support vector machine classifier. One essential part of a spinning machine is the bearing; hence, it is crucial to maintain the bearing’s health. A thorough comparison of the suggested approach with existing approaches was conducted using a benchmark-bearing dataset.

To reduce noise in both the frequency and time domains, Tian et al. [55] introduced the wavelet-SANet anti-noise, a wavelet-based self-attention network for machinery malfunction diagnostics. This approach combines frequency-oriented fusion modules and transformer modules. The experimental findings on two open-bearing datasets show good performance for identifying machine faults.

Wang et al. [56] used the dual-tree complex WPT with the sub-band averaging kurtogram to diagnose problems with spinning machinery. Their approach divides a signal into sub-signals using a sliding window, then the sub-band kurtosis is computed. The efficacy and advancements of the suggested method were validated by a simulation case and two applications for fault diagnosis of a planetary gearbox and a rolling bearing.

The normal multi-component signal produced by machinery vibration frequently has various interference components that obscure defective features. Zhang et al. [57] presented a weak feature augmentation method based on EWT and improved adaptive bistable stochastic resonance (IABSR) to extract faulty features in precision machinery. The approach achieved fault feature improvement in the low-frequency band of the harmonic spectrum by fully utilizing the signal decomposition capability of EWT and the signal enhancement of IABSR. These two case studies on the identification of machinery faults illustrated the usefulness and superiority of the suggested method.

Machine faults can be accurately diagnosed by using vibration signal properties such as instantaneous frequency, instantaneous amplitude, or spectral kurtosis. Shi et al. [58] developed a wavelet-based technique, dubbed wavelet-based synchro extracting transform (WSET), and applied it to fault diagnosis. Two rotor and rolling bearing benchmarks were used to test the efficacy of WSET in identifying failure features for malfunction identification.

An important subsystem of a high-speed train is the wheelset bearing system, and its service safety significantly depends on identifying and treating any compound problems in this system. In this sense, Ding [59] proposed a double impulsiveness measurement indices bilaterally driven EWT method to detect and diagnose defects. Additional demodulation was performed on the signals found in the sideband lower–upper boundary pairs of the EWT to find compound faults in the wheelset bearing system. Simulation, bench, and running tests validated the proposed method.

By analyzing the inter-harmonic content of the current signal, Gadanayak and Mallick [60] established a method for arcing high-impedance fault (HIF) identification in distribution feeders. The newly created unique knot-based empirical mode decomposition and maximum overlap discrete WPT was employed to separate the inter-harmonic components. The findings showed that the suggested method can detect HIFs quickly while achieving good security against failure.

Liu et al. [61] developed an approach to enhance EWT to address the spectrum segmentation flaw and improve the method’s capacity to extract bearing fault data. The maximum envelope-fitting method highlighted each mode and reduced the number of point extremes that were not useful. Reducing the number of filters suppresses noise interference on the modal. Data on gearbox bearing faults in wind turbines and locomotive bearings confirmed the method’s efficacy.

To fuse three-channel vibration signals for the weak failure detection of hydraulic pumps, Yu et al. [62] presented a novel vibration signal fusion approach combining the improved EWT and the variance contribution rate. Simulation and experiment analyses showed that the fusion method effectively detects weak faults in hydraulic pumps. From the literature, EWT has advanced the field of machine fault diagnostics.

While the above-mentioned related works employ various techniques to predict machine failures, our work differs by combining the strengths of both CNNs and LSTMs in a hybrid model. Using LSTM allows us to model patterns of the time series data. At the same time, CNN extracts important features such as trend changes and other patterns commonly observed in time series data, which are often variable.

The CNN-LSTM hybrid model has been successfully applied in many domains, including natural language processing and computer vision. Still, its application in time series forecasting, particularly in the context of predictive maintenance, has not been extensively explored. Our approach leverages these two architectures to identify potential anomalies. Additionally, we employ quantile regression to manage uncertainties present in the data. The approach enables us to make better predictions and identify potential anomalies, which may be challenging with traditional methods.

## 3. Dataset

The dataset is in time series format, collected from a sensor attached to the machine’s structure for use in this research. The machine consists of a three-phase, synchronous, alternating-current motor installed in an industrial plant with a vibration sensor attached to its casing. The motor is used in an exhaust fan located near a furnace with two poles and is powered by a frequency inverter that controls its speed. The findings can be evaluated for other types of equipment, such as hydraulic pumps, if vibration analysis is of interest.

The sensor is attached to the machine housing and can be either glued or bolted, ensuring proper contact to avoid noise in the data. The sensor measures the temperature on the surface of the housing and in the environment, rotation speed, frequency, vibrations, etc. For this research, only the vibrations in three axes, namely X, Y, and Z, are used as the input for the model, as they are relevant to detecting anomalies in the equipment, particularly in monitoring vibrations and imbalances.

After filtering and removing null values, the dataset comprised 7675 records between August 2021 and August 2022 (one year). It is worth noting that even though the data period is relatively long and vibration data is reported hourly, there are some periods within this interval where no data was collected for various reasons. Therefore, data pre-processing is necessary to generate more robust and reliable results.

**Figure 1:**
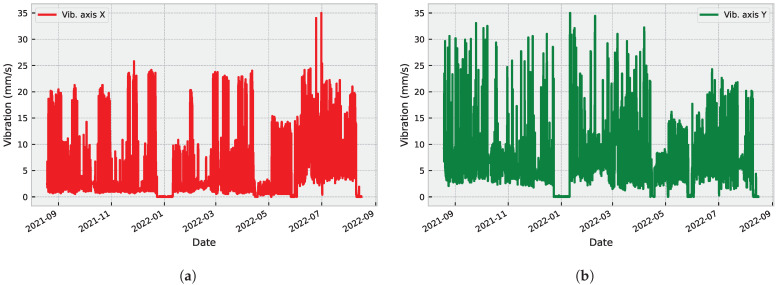

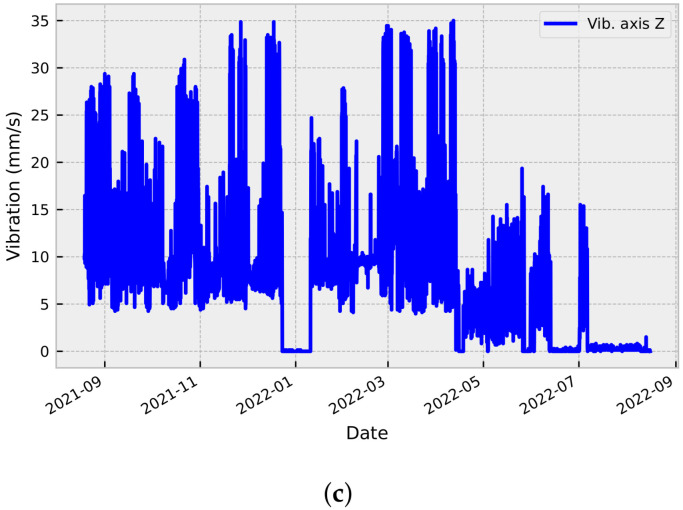
Raw vibration signals collected from three different axes: X (**a**), Y (**b**), and Z (**c**).

Figure 1 shows the raw vibration signals collected from three different axes: X (Figure 1a), Y (Figure 1b), and Z (Figure 1c). These signals display varying levels of amplitude and frequency, indicating different types of vibration patterns.

**Figure 2:**
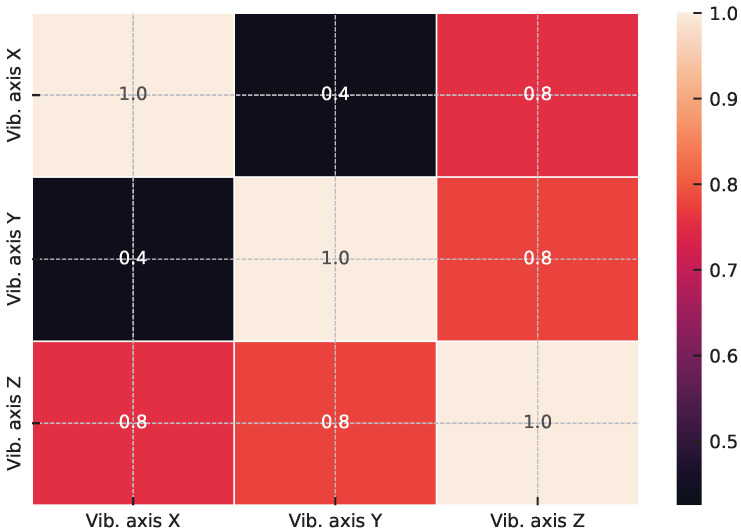
PhiK correlation matrix for the analyzed signals. The matrix shows the pair-wise correlations between signals, with warmer colors indicating stronger positive correlations and cooler colors indicating stronger negative correlations.

**Figure 3:**
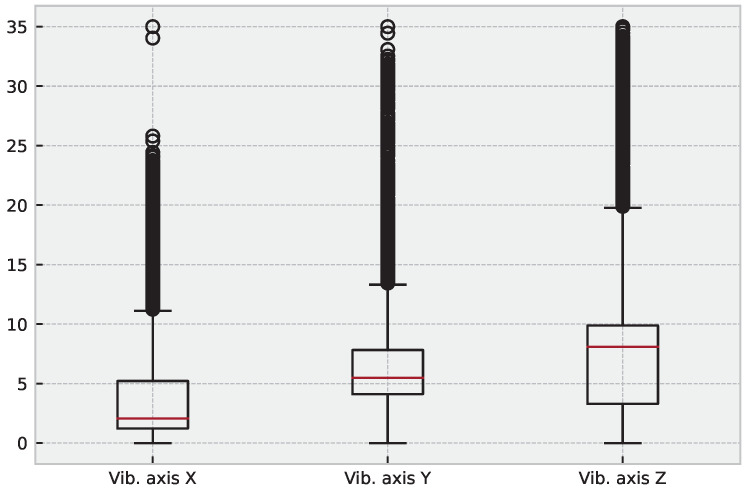
Boxplot for the analyzed signals. The plot displays the distribution of signal values, with each box representing the interquartile range (IQR) and the median line. The whiskers extend to the minimum and maximum values within 1.5 times the IQR, and any outliers beyond this range are shown as individual points.

Moreover, abnormal vibration records can lead to important conclusions about operating load, useful life, imbalance, and others. We present the dataset characteristics in Table 1 to further analyze the signals, and additional statistical characteristics of the considered dataset are presented in Figure 2 and Figure 3.

## 4. Methodology

This section will present the proposed method, along with a summary of the employed techniques: empirical wavelet transform, anomaly detection, and quantile regression will be explained in detail.

### 4.1. Empirical Wavelet Transform

The EWT is a signal processing technique that decomposes a signal into oscillatory modes with different scales and frequencies [63]. Given an input signal x(t) and a mother wavelet ψ(t), the EWT first generates a set of *n* non-linear and non-stationary functions called intrinsic mode functions (IMFs) using Algorithm 1 [64,65].
**Algorithm 1:** Empirical wavelet transform 
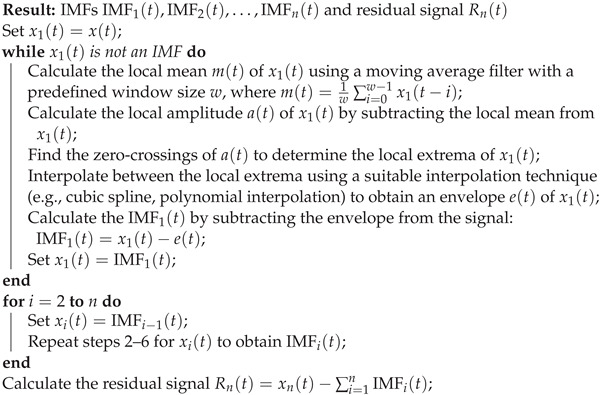


After obtaining the set of IMFs, the EWT applies a Fourier transform to each IMF to obtain a set of *n* spectrograms, which are used to visualize the time–frequency content of the signal. The EWT can be expressed mathematically as follows:

$$\begin{array}{cc} x(t) & =\sum_{i=1}^{n}{\mathrm{IMF}}_{i}\left(t\right)+R_{n}\left(t\right)\\ {\mathrm{IMF}}_{i}\left(t\right) & =\int x\left(\tau \right)h_{i}\left(\tau -t\right)d\tau \end{array}$$

where $h_{i}\left(\tau \right)$ is the *i*th filter defined as the convolution of the scaling function φ(t) and the mother wavelet ψ(t) scaled by a factor of $2^{i}$:

(1) $$h_{i}\left(\tau \right)=2^{i}\varphi \left(2^{i}\tau \right)\psi \left(2^{i}\tau \right)$$

A major advantage of EWT is its ability to adaptively decompose a signal into a set of components, each representing a distinct frequency band. This adaptability allows EWT to accurately capture a signal’s local and global characteristics, making it well-suited to analyze complex and irregular data patterns. EWT enables the extraction of information from signals with low signal-to-noise ratios [66].

The ability of EWT to handle non-stationary signals makes it a promising choice for analyzing time-varying data, such as those typically encountered in predictive maintenance tasks. Another benefit of EWT is its computational efficiency, important when working with large datasets or when real-time processing is required. Its flexibility in selecting wavelet functions allows for the optimal representation of the signal under analysis, further increasing its effectiveness in a wide range of applications [67].

#### Savitzky–Golay Filter

The Savitzky–Golay filter is a polynomial smoothing filter often used to remove noise from time series data while preserving the underlying trends in the data [68]. The filter works by fitting a polynomial of a specified order to a local window of the data and using this polynomial to estimate the smoothed values at each point in the time series [69].

Given a time series y(t) with *N* data points, the Savitzky–Golay filter estimates the smoothed value $\hat{y}\left(t\right)$ at each point using a polynomial of order *p* and a local window of size 2m+1:

(2) $$\hat{y}\left(t\right)=\sum_{k=-m}^{m}c_{k}y\left(t+k\right)\;\mathrm{for}\;m\leq t\leq N-m-1$$

where the coefficients $c_{k}$ are obtained by solving a least-squares problem that minimizes the sum of the squared errors between the polynomial fit and the original data:

(3) $$\min_{c_{k}}\sum_{k=-m}^{m}{\left(y\left(t+k\right)-\sum_{j=-m}^{m}c_{j}y\left(t+j\right)\right)}^{2}$$

The solution to this least-squares problem can be written in terms of a set of pre-computed coefficients that only depend on the order of the polynomial *p* and the size of the local window 2m+1. These coefficients can be pre-computed and stored in a matrix *M* for efficient computation of the smoothed values:

(4) $$\hat{y}=My$$

where $\hat{y}$ is a vector of length *N* containing the estimated smoothed values and y is a vector of length *N* containing the original data. The coefficients in the matrix *M* can be obtained as follows:

(5) $$M={\left(X^{T}X\right)}^{-1}X^{T}$$

where *X* is a matrix with dimensions (2m+1)×(p+1) containing the powers of the time variable *t* for the local window of size 2m+1 and the polynomial order *p*. Specifically, $X_{ij}=t_{i}^{j}$ for −m≤i≤m and 0≤j≤p.

### 4.2. Anomaly Detection

The ensemble of complementary outlier detection algorithms detects outliers in a dataset based on rare events in low-density regions of the probability distribution. The algorithm uses an ensemble of complementary detectors, each capturing a different aspect of outlier behavior. Formally, let $X_{1},X_{2},\ldots ,X_{n}$ be a set of *n* *d*-dimensional observations, where each observation $X_{i}=\left(X_{i1},X_{i2},\ldots ,X_{id}\right)\in R^{d}$ is a vector of *d* real-valued variables. The ECOD algorithm proceeds as follows:

For each variable j=1,2,…,d, the left and right tails of the empirical cumulative distribution functions (ECDFs) are estimated. Next, the ECOD computes the sample skewness coefficient for the *j*th feature distribution, used to determine whether to use the left- or right-tail probability in computing the outlier score.

An assessment of the observation is attained by computing three values: the O-left score, the O-right score, and the O-auto score. The O-left score constitutes an assessment of the outliers located in the lower tail of the distribution for each variable; the O-right score quantifies outliers situated in the upper tail of the distribution for each variable; and the O-auto score implements an adaptive adjustment of the tail probabilities based on the distribution’s skewness. The procedure is summarized in Algorithm 2.
**Algorithm 2:** ECOD outlier detection algorithm 
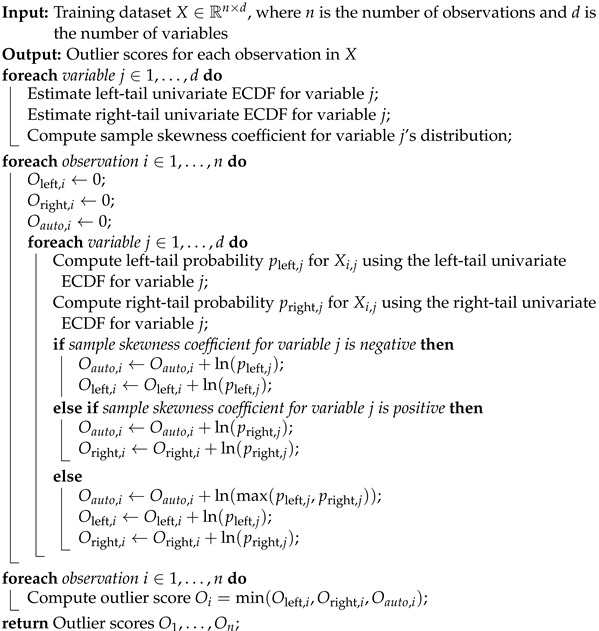


### 4.3. Quantile Regression

Quantile regression is an extension of the linear regression model that estimates the conditional quantiles of the response variable. It estimates the values of the response variable at various quantiles of the response’s conditional distribution given the predictor variables. The method is beneficial when the mean regression function does not represent the relationship between the response and predictor variables and when the response distribution is asymmetric or has heavy tails.

Let *Y* be the response variable and let $X=(X_{1},\ldots ,X_{p})$ represent a vector of *p* predictor variables. The quantile regression model can be formulated as follows:

(6) $$q_{\tau }\left(Y|X\right)=X^{T}\beta \left(\tau \right)+\epsilon_{\tau },$$

where $q_{\tau }\left(Y|X\right)$ is the conditional τ-quantile of *Y* given X, τ is the quantile level (with 0<τ<1), β(τ) is the vector representing the τ-quantile intercept and slope parameters, respectively, and $\epsilon_{\tau }$ is the error term that follows a τ-dependent distribution with zero mean and finite variance. The regression coefficients quantify the impact of the predictor variable on the τ-quantile of the response variable.

To estimate the quantile regression coefficients, one typically minimizes the following objective function:

(7) $$arg\min_{\beta }\sum_{i=1}^{n}\rho_{\tau }\left(y_{i}-x_{i}^{T}\beta \right),$$

where $y_{i}$ is the observed response for the *i*th observation, $x_{i}$ is the vector of predictor variables for the *i*th observation, β is the vector of quantile regression coefficients, and $\rho_{\tau }\left(u\right)=u\left(\tau -1u<0\right)$ is the check function. Here, 1u<0 is an indicator function that equals 1 when u<0 and 0 otherwise.

### 4.4. Limitations

A limitation in applying the proposed method is that the anomaly conditions can be related to high frequencies, and the use of filters can hide these patterns; therefore, an analysis of the relationship between identifying what is noise and what is a failure characteristic should be conducted.

## 5. Proposed Architecture

The proposed architecture for time series forecasting is a hybrid neural network that combines the strengths of LSTM and CNN. The network is designed to capture complex temporal dependencies in time series data by leveraging the complementary strengths of LSTM and CNN, while using attention mechanisms and gated residual units to improve the accuracy and stability of the predictions. Table 2 summarizes the main parameters and variables employed in this section.

First, the LSTM encoder processes the input sequence (given by the time series of interest) $X=(x_{1},x_{2},\cdots ,x_{T})$ to produce a sequence of hidden states $h=(h_{1},h_{2},\cdots ,h_{T})$, which summarize the temporal information of the input. The LSTM equations for computing the hidden states are:

(8) $$\begin{array}{cc} \; & i_{t}=\sigma \left(W_{xi}x_{t}+W_{hi}h_{t-1}+b_{i}\right) \end{array}$$

(9) $$\begin{array}{cc} \; & f_{t}=\sigma \left(W_{xf}x_{t}+W_{hf}h_{t-1}+b_{f}\right) \end{array}$$

(10) $$\begin{array}{cc} \; & o_{t}=\sigma \left(W_{xo}x_{t}+W_{ho}h_{t-1}+b_{o}\right) \end{array}$$

(11) $$\begin{array}{cc} \; & g_{t}=\tanh\left(W_{xg}x_{t}+W_{hg}h_{t-1}+b_{g}\right) \end{array}$$

(12) $$\begin{array}{cc} \; & c_{t}=f_{t}⊙c_{t-1}+i_{t}⊙g_{t} \end{array}$$

(13) $$\begin{array}{cc} \; & h_{t}=o_{t}⊙\tanh\left(c_{t}\right) \end{array}$$

in which σ is the sigmoid function, ⊙ is element-wise multiplication, and *W* and *b* are weight matrices and bias vectors, respectively. The input $x_{t}$ and hidden state $h_{t-1}$ are concatenated and multiplied by different weight matrices $W_{xi}$, $W_{hi}$, $W_{xf}$, $W_{hf}$, $W_{xo}$, $W_{ho}$, $W_{xg}$, and $W_{hg}$, as well as bias vectors $b_{i}$, $b_{f}$, $b_{o}$, and $b_{g}$, to produce input, forget, output, and candidate gate vectors $i_{t}$, $f_{t}$, $o_{t}$, and $g_{t}$. The output gate $o_{t}$ controls which part of the candidate memory cell $c_{t}$ is passed through the hyperbolic tangent activation function to produce the hidden state $h_{t}$ [70].

Next, an attention mechanism combines the encoder output *h* with the output of a CNN, denoted as *c*, which is a feature map of the input sequence obtained by applying convolutional filters to the time series. The attention mechanism computes a context vector $u_{t}$ as a weighted sum of the encoder output *h* and the CNN output *c*, where the weights are learned dynamically based on the final error through backpropagation. The attention weights $\alpha_{t,i}$ for each encoder hidden state $h_{i}$ and CNN output $c_{i}$ are computed as:

(14) $$\begin{array}{c} \alpha_{t,i}=\frac{\exp(s_{t}^{⊤}h_{i})}{\sum_{j=1}^{T}\exp\left(s_{t}^{⊤}h_{j}\right)} \end{array}$$

and the context vector $u_{t}$ is computed as follows:

(15) $$\begin{array}{c} u_{t}=\sum_{i=1}^{T}\alpha_{t,i}h_{i} \end{array}$$

After the attention mechanism combines the encoder output *h* and the CNN output *c* to produce the context vector $u_{t}$, the multi-head attention mechanism is used to link the decoder output $y_{t}$ to the hybridized LSTM output $u_{t}$.

The query matrix $Q_{t}$ corresponds to the decoder output at time step *t*, and has dimensions $d_{q}\times m$, where $d_{q}$ is the dimension of the query vector and *m* is the number of attention heads. The key and value matrices $K_{t}$ and $V_{t}$ correspond to the hybridized encoder and CNN output at time step *t*, respectively, and have dimensions $d_{k}\times T$ and $d_{v}\times T$, where $d_{k}$ and $d_{v}$ are the dimensions of the key and value vectors, respectively, and *T* is the length of the input time series. The multi-head attention weights $\beta_{t,i}$ for each key–value pair are then computed as follows:

(16) $$\begin{array}{c} \beta_{t,i}=\frac{\exp(Q_{t}^{h}K_{t,i})}{\sum_{j=1}^{T}\exp\left(Q_{t}^{h}K_{t,j}\right)} \end{array}$$

where $Q_{t}^{h}$ is the *h*th attention head of the query matrix $Q_{t}$, and $K_{t,i}$ is the *i*th column of the key matrix $K_{t}$. The multi-head context vector $v_{t}$ is then computed as a weighted sum of the value matrix $V_{t}$, using the attention weights $\beta_{t,i}$:

(17) $$\begin{array}{c} v_{t}=\sum_{i=1}^{T}\beta_{t,i}V_{t,i} \end{array}$$

where $V_{t,i}$ is the *i*th column of the value matrix $V_{t}$.

After the multi-head attention mechanism links the decoder output to the hybridized encoder and the CNN output, the output is passed through gated residual networks (GRN) to produce the final quantile regression outputs.

Specifically, the GRN takes the multi-head context vector $v_{t}$ as the input and first applies two separate linear transformations, denoted as $W_{1}$ and $W_{2}$, to the input vector $v_{t}$. The resulting output is then passed through a Gaussian error linear unit (GELU) activation function, followed by another linear transformation, denoted as $W_{3}$, to produce the intermediate output $\eta_{2}$:

(18) $$\begin{array}{cc} \eta_{1} & =\mathrm{GELU}(W_{1}v_{t}+W_{2}c_{t}+b_{1}) \end{array}$$

(19) $$\begin{array}{cc} \eta_{2} & =W_{3}\eta_{1}+b_{2} \end{array}$$

where $b_{1}$ and $b_{2}$ are bias terms; and the GELU activation function is given by:

(20) $$\begin{array}{c} \mathrm{GELU}(x)=x\Phi (x), \end{array}$$

where Φ(x) is the cumulative distribution function (CDF) of the standard normal distribution, i.e.,

(21) $$\begin{array}{c} \Phi \left(x\right)=\frac{1}{2}\left(1+\mathrm{erf}\left(\frac{x}{\sqrt{2}}\right)\right). \end{array}$$

The GELU function applies the identity function to positive inputs and smoothly maps negative inputs to zero, using the CDF of the standard normal distribution to introduce non-linearity. The resulting function is continuous and differentiable everywhere [71].

The intermediate output $\eta_{2}$ is then passed through the gated linear unit (GLU) transformation, allowing for the suppression of unnecessary parts of the GRN. The GLU transformation is defined as follows:

(22) $$\begin{array}{cc} \mathrm{GLU}(\eta_{2}) & =\sigma \left(W_{4}\eta_{2}+b_{4}\right)⊙\left(W_{5}\eta_{2}+b_{5}\right) \end{array}$$

where σ is the sigmoid activation function, and $W_{4}$, $W_{5}$, $b_{4}$, and $b_{5}$ are learned parameters.

**Figure 4:**
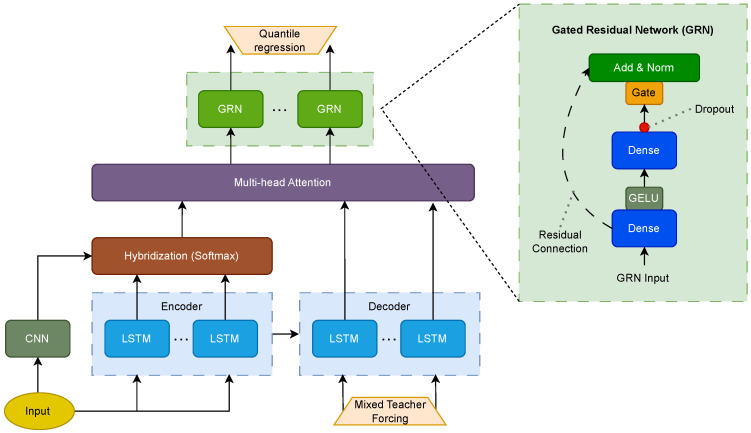
Graphical representation of the hybrid LSTM-CNN with attention and GRN for time series forecasting.

Finally, the output of the GLU transformation is added to the input vector $v_{t}$ and passed through layer normalization to produce the final quantile regression output ${\hat{y}}_{t}$:

(23) $$\begin{array}{cc} {\hat{y}}_{t} & =\mathrm{LayerNorm}(v_{t}+\mathrm{GLU}\left(\eta_{2}\right)) \end{array}$$

where layer normalization helps to stabilize network training. The procedure is summarized in Algorithm 3. The structure of the proposed method is shown in Figure 4.
**Algorithm 3:** Hybrid LSTM-CNN with attention and GRN for time series forecasting 
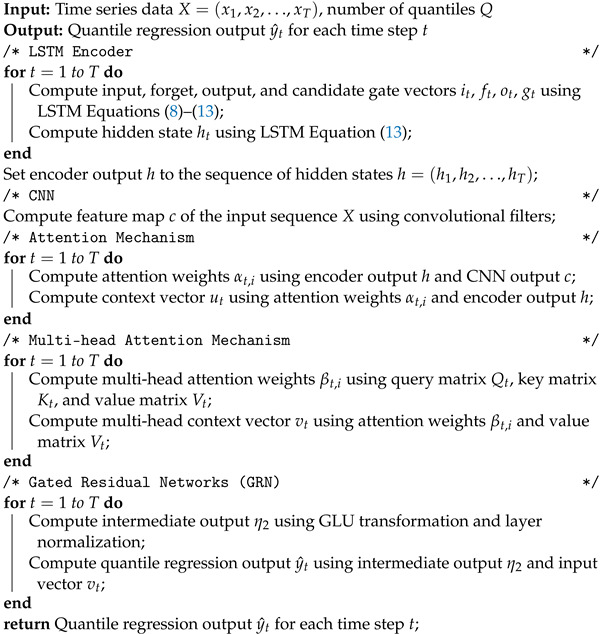


## 6. Results

In this study, the time series signals were pre-processed using several techniques to enhance the accuracy of the forecasting model. This step is considered crucial, since time series data may have characteristics that can affect forecasting models, such as noise, missing values, and seasonality. Data pre-processing improves model accuracy and interpretability.

Data noise can arise from measurement errors, recording inconsistencies, or random fluctuations. Smoothing techniques, such as moving averages or exponential smoothing, can reduce the impact of noise and improve model pattern capture. Missing values in time series data can lead to gaps in the input sequence, resulting in poor predictions.

Thus, we pre-processed the data to ensure the input sequences were clean and suitable for our hybrid LSTM-CNN architecture. Concerning this, the signals were first normalized using min–max normalization to ensure that all data points fell within the same range, thereby preventing the influence of outliers on the model.

**Figure 5:**
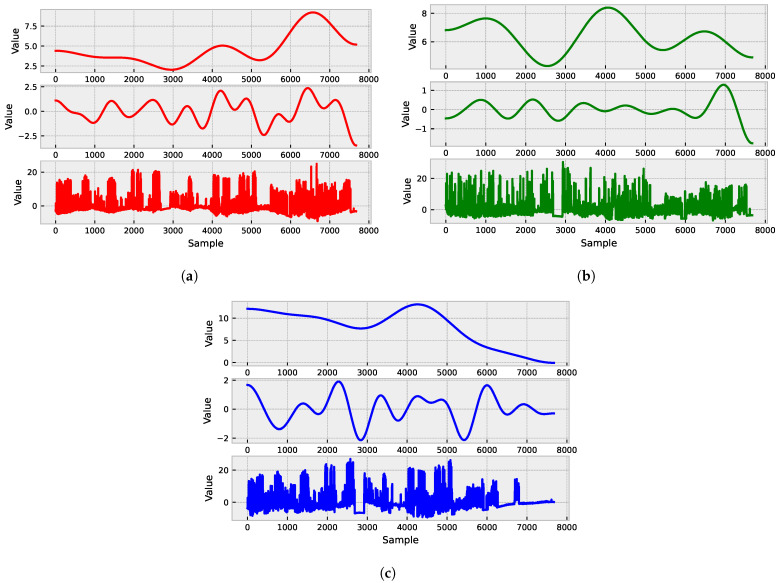
EWT decomposition of the signal captures any trends in the low-frequency components and allows for the de-trending of the signals to remove long-term patterns or irregularities that could affect the model’s accuracy. Resulting EWT decomposition for axes: X (**a**), Y (**b**), and Z (**c**).

To capture the trend of the signals, we utilized the EWT (see Figure 5),to decompose the signal into different frequency bands and capture any trends in the low-frequency components. This allowed us to de-trend the signals and remove any long-term patterns or irregularities that could affect the model’s accuracy.

**Figure 6:**
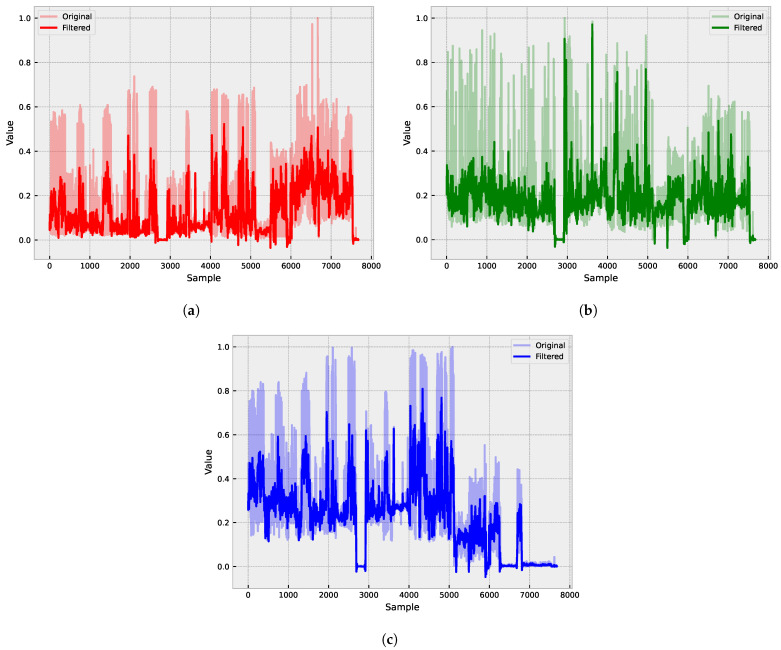
Application of a smoothing filter to improve signal quality. The Savitzky–Golay filter removes high-frequency noise and fluctuations while preserving the signal’s shape and trend. This results in a cleaner and more accurate signal that can be used as input for the forecasting model. Original and filtered signals for axes: X (**a**), Y (**b**), and Z (**c**).

Finally, we applied a Savitzky–Golay to filter the signals and remove any remaining high-frequency noise or fluctuations. Figure 6 presents the impact of applying the Savitzky–Golay filter to the time series data. The figure includes two plots: one displaying the original, noisy signal and the other showing the filtered signal after applying the Savitzky–Golay filter. The plots are designed to visually demonstrate the effectiveness of the filter in reducing high-frequency noise while preserving the overall shape and trend of the original signal. By comparing the two plots, it becomes clear that the filtered signal is smoother and less affected by noise, making it a more suitable input for the forecasting model.

### 6.1. Time Series Forecasting

First, we compared the proposed model with traditional regression methods available from most off-the-shelf forecasting libraries. We relied on the traditional MSE (mean square error) metric, as shown in Table 3. The table shows that the proposed model outperforms the analyzed regression methods. The following configuration was used: a sequence of 50 input time steps to predict the next 5 time steps, resulting in a network with 11,343,653 trainable parameters. A one-layer LSTM was used both in the encoder and decoder, each with 64 hidden units; while the multi-head attention mechanism was set to 16 heads. For the CNN, a pre-trained ResNet18 was employed.

Then, Table 4 displays the performance of three different models regarding quantile regression (QR) accuracy for the 10% and 90% percentiles. The first model listed is the default Seq2Seq model, which serves as a baseline for comparison. The second model, Seq2Seq + MHA, includes a multi-head attention mechanism to improve the accuracy of the predictions. Finally, the third model is the proposed model, which utilizes the hybrid LSTM-CNN architecture with attention and gated residual networks (GRN) to enhance the accuracy of the predictions.

The results show that both the Seq2Seq + MHA and proposed models outperform the default Seq2Seq model for both quantiles, with the proposed model achieving the lowest QR values of 0.0031 and 0.0030 for the 10% and 90% percentiles, respectively. This indicates that the proposed model is more accurate in predicting extreme events in the time series data.

**Figure 7:**
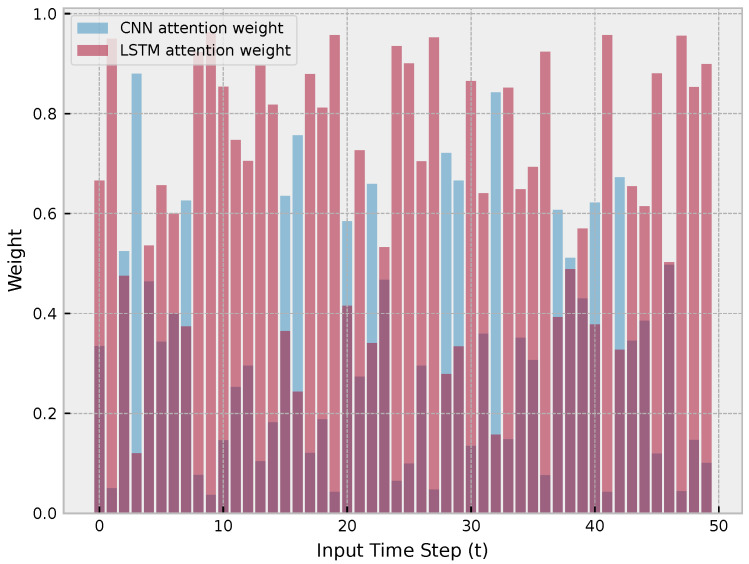
Illustration of how the network dynamically adjusts the weights of the LSTM encoder and CNN input to improve the results.

The findings of this study suggest that the hybrid LSTM-CNN architecture with attention and GRN is an effective approach for time series forecasting, particularly when predicting extreme events. The results also highlight the importance of utilizing attention mechanisms and GRN to enhance the accuracy of the predictions. Figure 7 illustrates the effectiveness of the hybrid LSTM-CNN architecture with attention and GRN for time series forecasting. The figure displays the way in which the network combines the inputs from the LSTM encoder and the CNN input to generate improved predictions, particularly for extreme events.

**Figure 8:**
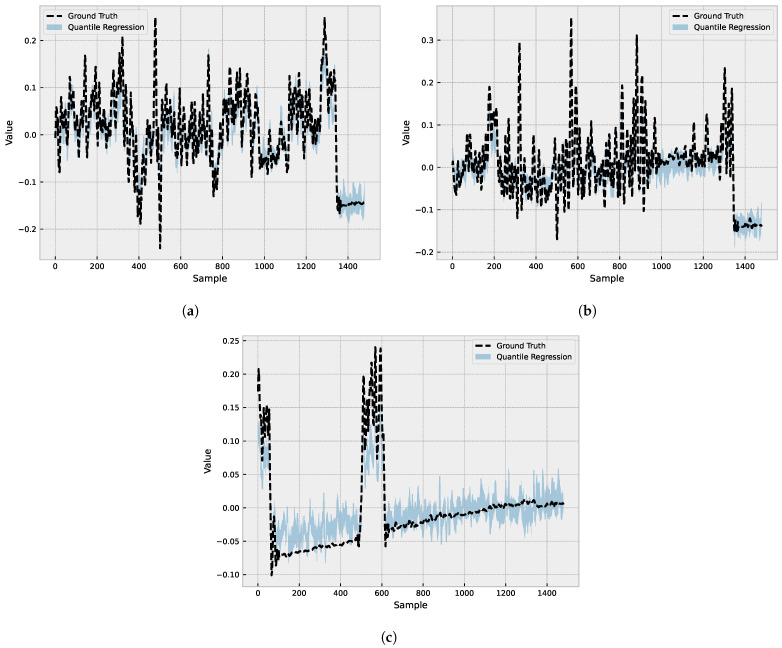
The figures show the predicted values for the 10% and 90% quantile ranges (QR) for the X (**a**); Y (**b**); and Z (**c**) axes. The shaded area between the QRs represents the range of values that contain 80% of the predicted values.

Figure 8 displays the results of three different predictions made by the forecasting model. Each figure presents a plot displaying the actual values (shown as a dashed line) along with the 10% and 90% quantile ranges (QR) illustrated as two separate lines. The shaded area between the QR lines represents the range of values that contain 80% of the predicted values. By visually analyzing the shaded area in relation to the actual values, we can assess the model’s accuracy and its ability to capture the range of possible outcomes. A narrow shaded area indicates that the model is more confident in its predictions, while a wider shaded area signifies greater uncertainty.

### 6.2. Anomaly Detection

**Figure 9:**
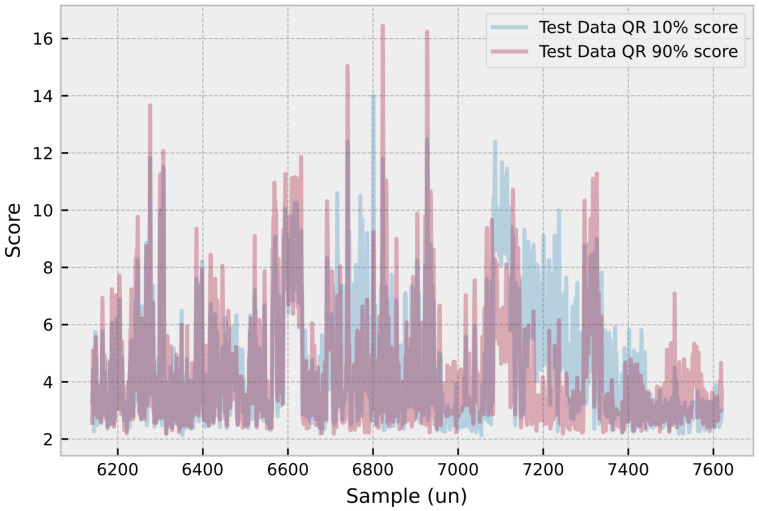
ECOD algorithm leverages quantile predictions for probability-based maintenance planning.

Figure 9 demonstrates the application of the ECOD anomaly detection algorithm in the context of the neural-based predictive maintenance tool for electrical machines. The figure presents a plot that combines the 10% and 90% quantile predictions with the anomaly detection results derived from the ECOD algorithm. The plot showcases how the algorithm identifies potential faults within the given quantile range, allowing for a more comprehensive assessment of the machine’s health. By combining the quantile predictions with ECOD anomaly detection, machine operators can gain a deeper understanding of the machine’s health and the probability of faults occurring. This information enables them to make more informed decisions regarding maintenance planning and take proactive measures to address potential issues.

## 7. Conclusions

In conclusion, this research addresses the critical issue of predictive maintenance for electrical machines. This study provides a neural-based predictive maintenance tool by developing a custom hybrid CNN-LSTM attention model utilizing quantile regression. This tool can effectively predict electrical machine failures and manage uncertainties present in the data.

Using vibration sensor data measured in three axes (axial, radial, and radial X) and applying advanced neural network techniques provides an accurate and efficient predictive maintenance tool that can greatly benefit companies. The developed tool allows companies to optimize their maintenance schedules and improve the overall performance of their electrical machines, ultimately reducing maintenance costs, increasing efficiency, and minimizing unplanned downtime.

Once the proposed model is properly trained, it can be used with inference data to define which equipment is most likely to fail concerning the machine’s vibration characteristics. This analysis can be used in predictive maintenance, providing more information about the machine’s health under evaluation.

While the data employed in this analysis is derived from a three-phase motor, the model has the potential to be expanded to other equipment with similar operations. Future work should be conducted applying the method in the field and determining its efficiency in automatically identifying equipment that needs maintenance based on machine learning, thus making the operators’ tasks easier.

## Figures and Tables

**Table 1:** Dataset characteristics.

	Vib. Axis X	Vib. Axis Y	Vib. Axis Z
Mean	4.5519	6.3701	7.9366
Median	2.0588	5.4902	8.0980
Mode	1.3725	0.0000	0.1373
Range	35.0000	35.0000	35.0000
Variance	29.8170	20.0177	46.2006
Std. Dev.	5.4605	4.4741	6.7971
25th %ile	1.2353	4.1176	3.2941
50th %ile	2.0588	5.4902	8.0980
75th %ile	5.2157	7.8235	9.8824
IQR	3.9804	3.7059	6.5882
Skewness	1.7963	2.3620	1.4814
Kurtosis	2.4265	8.7628	3.0811

**Table 2:** Summary of parameters and variables in the proposed architecture.

Symbol	Description
*X*	Input sequence (time series of interest)
$x_{t}$	Input at time step *t*
*h*	Sequence of hidden states from LSTM encoder
$h_{t}$	Hidden state at time step *t*
*W*	Weight matrices
*b*	Bias vectors
$i_{t}$	Input gate vector at time step *t*
$f_{t}$	Forget gate vector at time step *t*
$o_{t}$	Output gate vector at time step *t*
$g_{t}$	Candidate gate vector at time step *t*
$c_{t}$	Candidate memory cell at time step *t*
*c*	Feature map of the input sequence from CNN
$\alpha_{t,i}$	Attention weights for encoder hidden state $h_{i}$ and CNN output $c_{i}$
$u_{t}$	Context vector at time step *t*
$Q_{t}$	Query matrix at time step *t*
$K_{t}$	Key matrix at time step *t*
$V_{t}$	Value matrix at time step *t*
$\beta_{t,i}$	Multi-head attention weights for key-value pairs
$v_{t}$	Multi-head context vector at time step *t*
$\eta_{1}$	Intermediate output of the GRN
$\eta_{2}$	Intermediate output of the GRN
${\hat{y}}_{t}$	Quantile regression output at time step *t*

**Table 3:** Comparison of the proposed model with the traditional benchmarks regarding the MSE.

Model	MSE
ElasticNet Regression	0.0056
Decision Tree Regressor	0.0048
RandomForestRegressor	0.0023
K-Nearest Neighbours Regressor	0.0051
XGBoost	0.0013
Bagging Regressor	0.0025
Extra Trees Regressor	0.0020
MLP Regressor	0.0015
Gaussian Process Regressor	0.0350
Proposed Model	0.0006

**Table 4:** Comparison of the proposed model with the traditional benchmarks regarding the QR.

Model	QR 10%	QR 90%
Default Seq2Seq	0.0056	0.0068
Seq2Seq + MHA	0.0056	0.0064
Proposed Model w/ResNet18	0.0028	0.0030

## Data Availability

The dataset used in the experiments of this paper is confidential.
